# Recent Strategies for Detection and Improvement of Brown Planthopper Resistance Genes in Rice: A Review

**DOI:** 10.3390/plants9091202

**Published:** 2020-09-14

**Authors:** Bello Sani Haliru, Mohd Y. Rafii, Norida Mazlan, Shairul Izan Ramlee, Isma’ila Muhammad, Ibrahim Silas Akos, Jamilu Halidu, Senesie Swaray, Yusuf Rini Bashir

**Affiliations:** 1 Laboratory of Climate-Smart Food Crop Production, Institute of Tropical Agriculture and Food Security, Universiti Putra Malaysia, UPM Serdang 43400, Selangor, Malaysia; bellosanihaliru@gmail.com (B.S.H.); ismuha2000@gmail.com (I.M.); akosibrahims@gmail.com (I.S.A.); jamiluhalidu@gmail.com (J.H.); 2 Department of Crop Science, Usmanu Danfodiyo University, Sokoto P. M. B. 2346, Sokoto State, Nigeria; 3 Department of Crop Science, Faculty of Agriculture, Universiti Putra Malaysia, UPM Serdang 43400, Selangor, Malaysia; shairul@upm.edu.my (S.I.R.); senesieswaray74@gmail.com (S.S.); yusufrinibashir@gmail.com (Y.R.B.); 4 Department of Agriculture Technology, Faculty of Agriculture, Universiti Putra Malaysia, UPM Serdang 43400, Selangor, Malaysia; noridamz@upm.edu.my

**Keywords:** brown planthopper, rice, resistance gene, quantitative trait loci, gene mapping, marker-aided selection

## Abstract

Brown planthopper (BPH; *Nilaparvata lugens* Stal) is considered the main rice insect pest in Asia. Several BPH-resistant varieties of rice have been bred previously and released for large-scale production in various rice-growing regions. However, the frequent surfacing of new BPH biotypes necessitates the evolution of new rice varieties that have a wide genetic base to overcome BPH attacks. Nowadays, with the introduction of molecular approaches in varietal development, it is possible to combine multiple genes from diverse sources into a single genetic background for durable resistance. At present, above 37 BPH-resistant genes/polygenes have been detected from wild species and indica varieties, which are situated on chromosomes 1, 3, 4, 6, 7, 8, 9, 10, 11 and 12. Five BPH gene clusters have been identified from chromosomes 3, 4, 6, and 12. In addition, eight BPH-resistant genes have been successfully cloned. It is hoped that many more resistance genes will be explored through screening of additional domesticated and undomesticated species in due course.

## 1. Introduction

Rice (*Oryza sativa* L.) is a vital nourishment for the world’s teeming population [1]. In terms of production worldwide, it is ranked third after sugarcane and maize [2]. Among the rice insect pests, the brown planthopper (BPH) has been regarded as one of the main rice production constraints in Asia [3,4,5,6]. Currently, the insect pest has been described as the most damaging rice pest in Asia [5,6,7,8]. Heavy infestation by BPH can result to direct and indirect yield losses of 20–80% in sensitive cultivars [9,10,11,12,13]. When the pest damage becomes established, it is very difficult to control and, thus, yield reduction is certain [14]. The factors that favour BPH development include the adoption of high-yielding BPH-sensitive varieties to replace traditional resistant ones in recent years [15], higher humidity (greater than 80%), temperature of about 25 to 32 °C, and application of nitrogenous fertiliser beyond the recommended rate [16]. Others are higher plant density, continuous submergence of the host in the field and indiscriminate application of pesticide at the early development of the host, which destroys their natural enemies [12,16,17]. Attack by BPH causes a reduction in photosynthetic rate, leaf area, chlorophyll content, nitrogen level of leaf and stem and dry matter accumulation in rice varieties that are susceptible [18].

In China, Korea, Vietnam and Japan, severe damage was recorded due to BPH attack. In China alone, yield losses of 2.7 million tonnes of rice in 2005 and 2008 were attributed to attack by BPH. For instance, BPH-transmitted diseases, such as ragged-stunt virus and grassy-stunt virus of rice, led to a yield reduction of 400,000 tonnes of rice in Vietnam [19]. In Malaysia, BPH infestation has been on the increase, and, presently, more hopperburn cases have been recorded [20]. Outbreaks of BPH in the early 20th century, particularly in South-East Asian countries, has drawn the attention of scientists, especially plant geneticists and breeders, to devise various effective and affordable means to overcome the devastating effect of the pest [21]. In addition, genetic knowledge of BPH resistance and its population dynamics are important for plant breeders when deciding on the appropriate breeding strategies to be deployed [18]. To curtail BPH attack, various management strategies have been utilised in Asian countries [13]. Frequent usage of chemicals to overcome BPH attack can result in deleterious consequences to the pests’ natural enemies [17], causing toxicity to the environment and increasing the cost of production and health hazards to humans [22]. Development of resistant varieties of rice should be the main priority of plant breeders in order to substitute traditional BPH management strategies [23]. This is because genetic resistance remains, economically, the most viable alternative health-wise and an environmentally safe management strategy for BPH control [22,24].

Brar et al. [19] and Fujita et al. [25] have produced review articles of BPH-resistant gene loci alongside other hoppers found in Asia. Similarly, Jena and Kim [4] and Zhang [26] have documented review papers on the genetics of BPH resistance and breeding in rice. In the same vein, Hu et al. [27] recently reviewed the advancement made so far on molecular breeding and genetics of BPH-resistant gene loci in rice. All the above previous reviewers have made significant contributions in providing substantial information about BPH-resistant gene loci discovered up until their research [25]. However, many more gene loci have been documented since then [13,28,29,30,31,32,33,34], and there is a need to update the rice scientific community on the journey made so far. This will help the scientists in no small way when planning their BPH breeding programme. In our present review appraisal, we provide an update on mapping and identifying quantitative trait loci (QTLs), gene cloning, and gene clusters, among others.

## 2. Rice Mechanisms for BPH Resistance

Various resistance mechanisms of rice to curtail BPH attack include antibiosis, antixenosis and tolerance (Table 1) [35]. The most widely studied defensive mechanism in rice is antibiosis [36,37,38]. Antibiosis is a resistance mechanism by which the host plant inflicts injury or mortality, decreased growth or longevity, as well as a reduction of pest reproduction. [39]. Antixenosis or nonpreference, on the other hand, refers to the resistance mechanism whereby the host plant exhibits features that are unattractive or desirable for the insect pest to invade or damage. Some of these features include colour, odour, and texture. Tolerance is a resistance mechanism by which plant is able to recover or withstand insect pest damage and, in addition, produce well despite manifesting infestation symptoms [39,40]. Many investigators have reported antibiosis, antixenosis, as well as tolerance in rice germplasm used for the improvement of BPH-resistant genes [20,23,29,30,33,41,42].

## 3. Genetics and Improvement of Rice for BPH Resistance

The discovery of a BPH-resistant gene source was first established in 1967 [44]. Similarly, Athwal et al. [43] discovered *BPH1* and *BPH2* resistance loci in Mudgo and ASD7, respectively. Additional loci for resistance, such as *BPH3* and *BPH4,* were found through genetic analysis of other donors [45,46]. Subsequently, several resistant donors (Table 1) for diverse BPH populations were derived from cultivated and wild species. The donors include ARC10550, Swarnalata, Pbt33, T12, Balamawee, Rathu Heenati, Chin Saba and Babawee. Others are *O. minuta*, *O*. *officinalis*, *O. australiensis* and *O. latifolia* [4]. In most breeding programmes, particularly in South-East Asia, four resistant loci (*BPH4*, *BPH2*, *BPH3* and *BPH1*) have been widely utilised [47]. Consequently, the International Rice Research Institute (IRRI) has officially released many resistant varieties to farmers in Asia for commercial cultivation. However, improved varieties possessing these genetic loci missed their BPH resistance due to new biotype emergence. The first resistant variety of rice (IR26) developed by IRRI, which possesses the *BPH1*-resistant locus, became sensitive to BPH because of the emergence of biotype 2 in 1976–1977. A *BPH2*-resistant locus discovered earlier was introgressed into rice cultivars to overcome BPH biotype 2 [48]. The resistance to BPH in IR36 was sustained for a period of two years [35]. Many varieties with the *BPH2* locus were extensively cultivated in Vietnam, Indonesia and the Philippines [4]. Later, another biotype (biotype 3) emerged, which rendered the resistance of *BPH2* obsolete. Subsequently, *BPH3* and *BPH4* resistant loci were introgressed into higher-yielding rice cultivars to curtail attack by BPH [45]. Varieties such as IR56 and IR60 were released by the IRRI in 1982 in the Philippines. These varieties have *BPH3-*derived resistance from Rathu Heenati, a Sri Lankan traditional cultivar. Another variety that has a *BPH4* resistant locus, IR66, was developed and released to farmers for estate cultivation in 1987. Other varieties such as IR68, IR70, IR72 and IR74, which also possess a *BPH3*-resistant locus, were released in 1988, and they have all established resistance to biotype 3 [4].

In a screening experiment of about 3300 genotypes and breeding lines across the globe in Japan, Kaneda et al. [49] revealed that the majority of the resistant landraces were from the southern states of India and Sri Lanka. Based on the reaction of these varieties to various biotypes of BPH, 60% of Sri Lankan cultivars have the *BPH2* locus. On the contrary, merely 10% of the cultivars from India possess the locus. However, in most of the research conducted, untamed species of rice showed a more wide-resistance spectrum to BPH than the cultivated traditional varieties [50]. Many rice accessions were screened for resistance against three biotypes of BPH at the IRRI. Out of 44,335 rice accessions that were tested for resistance to BPH biotype 1, only 15.4% were resistant. For BPH biotype 2, 10,553 were evaluated, but just 1.9% were resistant, while for BPH biotype 3, 13,021 were screened, but only 1.8% were resistant [51].

In genetic studies of 20 BPH-resistant varieties of rice, Sidhu and Khush [52] reported that two genes controlled the resistance in three varieties, seven varieties had *BPH3*, and 10 varieties possessed the *BPH4*-resistant locus. The *BPH3*-resistant locus acquired from Rathu Heenati segregates independently of *BPH1*. Similarly, *BPH4*, which is the gene locus for resistance in the variety Babawee, segregates independently from *BPH2*. Athwal et al. [43] made a comparison between resistant varieties and reported that the *BPH1* locus governed the resistance in Mudgo, CO22 and MTU15. Another investigation discovered that the *BPH1* locus has a close relationship with *BPH2,* and no reunion has been established between them [4]. The MGL2 rice variety carries a *BPH1* locus, while the Ptb18 variety carries a *BPH2* locus [53]. In a linkage analysis of *BPH3* and *BPH4* loci, Sidhu et al. [54] reported that these genes are closely linked. Another study reported that the semidwarf recessive gene (*sd1*) is related to the *BPH4* locus. The *BPH1* and *BPH2* resistant loci were found to segregate independently from *BPH3* and *BPH4* [55]. However, Ikeda and Kaneda [56] revealed that *BPH2* and *BPH1,* as well as *BPH3* and *BPH4,* are closely associated. In a trisomic analysis of *BPH3* and *BPH4*, it was discovered that the genetic loci of these two genes were located on chromosome 10 [55]. Khush et al. [46] observed that *bph5* locus was detected from cultivar ARC10550. Further genetic analysis reveals that *bph5* separated freely from *BPH4*, *BPH1*, *BPH2* and *BPH3*. In a genotypic assessment of traditional rice cultivars using four BPH biotypes, Khush [57] recorded nine major genes that confer BPH resistance. Four of the resistance genes were dominant, and five were recessive. Khush [58] revealed that rice cultivars such as Pokkali, Kaharamana and Balamawee possess single dominant BPH-resistant genes that are allelic to each other. Murata et al. [59] disclosed that the dominant gene that confers BPH resistance in Balamawee, Karahamana and Pokkali was termed *Bph9.* In addition, these three cultivars resist attack from BPH biotypes 1, 2 and 3. In an allelism test and fine genetic studies of rice cultivars Col. 5 and Col. 11, derived from Thailand, Nemato et al. [60] confirmed that the recessive gene in these two cultivars differed from *bph5* and *bph7* and, thus, it was named *bph8*. Additional genetic analysis shows that each of these two varieties possesses single recessive BPH-resistant genes that are allelic to each other, i.e., one gene does not complement another [58]. The *BPH3* resistance locus peculiar to Ptb33 and Rathu Heenati is closely associated with *BPH4* in Babawee [61]).

Nowadays, marker-aided selection (MAS) has been widely applied as a very vital instrument for the identification and introgression of BPH resistance genes into susceptible rice cultivars [33,62,63,64]. Molecular markers are now becoming very important tools for the incorporation of desired genes and for the determination of polymorphisms between parental lines intended for the MAS programme [65]. Application of molecular markers facilitates and hastens the desired gene introgression processes due to the fact that it saves time on mass or phenotypic selection, reduces the costs involved and accords more reliability to the selection result, which is free from the effect of environmental actors [66,67]. Several molecular markers have been adopted for the detection and introgression of genes that accord BPH resistance in rice, and they are sequence-tagged sites (STSs) [38,62], simple sequence repeats (SSRs) [32,64,68,69,70,71,72], amplified fragment length polymorphisms (AFLPs) [63,73], single nucleotide polymorphisms (SNPs) [13,31,74], rapid amplified polymorphisms DNAs (RAPDs) [62,75], rapid fragment length polymorphisms (RFLPs) [63,73,76,77], and insertion deletions (InDels) [28,33,34,78,79].

The marker-aided backcrossing (MAB) strategy has been widely adopted for the introgression of genes that improve resistance to BPH in rice cultivars [3,7,80]. The idea behind MAB is to reduce the genome content of the pollen parent into the genetic make-up of the recipient parent [65]. The fundamental prerequisite of MAS is the discovery of tightly linked/functional markers through linkage analysis. Furthermore, the selection of desirable gene stems from the position of the marker and space or distance between the gene of interest and the marker [65]. Various genes that administer resistance to BPH have been successfully transferred into BPH-susceptible cultivars through MAB [5,68,81,82,83,84,85,86,87].

In recent years, the marker-aided gene-pyramiding approach has received attention to improve the resistance of elite rice cultivars to BPH [88,89,90]. This is because combining multiple genes in single genetic background hinders insect pest adaptability to rice cultivars and, thus, improves the durability of resistance [91]. The prosperity of any gene-pyramiding approach stems from many considerations, such as the nature of genetic materials, the number of genotypes chosen in each generation of breeding, the interval between the flanking markers and desired gene and the number of target genes to be chosen [92]. Many authors have adopted the gene-pyramiding approach in order to improve the BPH resistance of rice cultivars [81,89,90,92,93,94].

## 4. Methods for BPH Screening in Rice

The screening of rice germplasm for BPH resistance started as far back as 1967 when sources of BPH biotype resistance were first recorded [44]. Since then, various methods have been employed at the IRRI for the screening of germplasm. Fujita et al. [25] reported that the standard seed-box screening test (SSST) is the most widely used mass screening method for phenotyping in leafhoppers and planthoppers. The mass screening method that has been widely used by the IRRI and the National Agricultural Research and Extension System (NARES). This method involves evaluating the genotypes at the juvenile stage (when the seedlings produce two or three leaves) when BPH nymphs are at second instars using a seed-box in a screen-house, i.e., the modified seed-box test [95]. The screening method, through the seed-box test in the screen house, utilises nymphs of BPH with independent selection of plant substances at the young (seedling) stage and, sometimes, the screening is spread across various developmental stages of plant growth. Furthermore, the modified seed-box test is utilised to assess seedling damage symptoms by the progenies of the first nymphs [96]. Another mass screening method is the natural field population whereby the host is subjected to natural field infestation, especially in BPH endemic areas, to assess genotype resistance and susceptibility. However, the major drawbacks of field screening are the unpredictability and non-uniformity of BPH distribution, the seasonality of BPH and rendering field screening unreliable [4]. In order to economise time and space, the seed-box method is the most widely adopted method, with free choice to find out the genotypes that are resistant or susceptible in the screen-house [97]. However, irrespective of evaluation technique, absolute care should adequately be observed to ensure the purity of BPH insects to be used for evaluation. Furthermore, the mechanism of resistance by antibiosis can be detected with no choice of the insect pest using the restructured or modified seed-box test. Thus, it is essential to determine resilience to BPH via the seed-box test inside the screen-house, applying the independent selection method (i.e., antixenosis), and in the field to physically confirm the resistance and susceptibility of genotypes [4,98].

## 5. Mapping and Identification of QTLs

QTLs/polygenes are controlled by many genes and the environment plays an important role in their expression [40,65,99]. Examples of quantitative traits are yield, resistance to diseases and pests, and drought. QTLs mapping can be achieved in three basic ways, i.e., standard interval mapping, composite interval mapping and single-marker analysis [100]. Single-marker analysis is a method by which the mapping population is categorised into groups at each marker locus based on phenotype. QTLs can be detected if the differences are significant in the total average phenotypic score for each group [101]. In standard interval mapping, QTLs can be more easily mapped than with single-marker analysis. Here, a flanking marker is employed at the interval of two markers at every locus. The major shortcoming of this method is the detection of incorrect QTLs due to linked and unlinked QTLs. Composite interval mapping is the most frequently used strategy for QTLs mapping. In this method, fraction of markers are applied at both linked and unlinked QTLs. This method is also used to determine the interaction between QTLs detectors [102].

As reported in the literature so far, above 37 genes/QTLs that administer BPH resistance have been discovered [13,25,27,29,30,78,79,103] These genes have been designated to different locations on chromosomes 1, 3, 4, 6, 7, 8, 9, 10, 11 and 12 [104,105,106]. In addition, five BPH resistance gene clusters (Table 2) have been reported from four (3, 4, 6 and 12) of the ten chromosomes [13,27]. Basically, a gene cluster refers to a combination of two or more genes that are in close proximity to each other in similar chromosomal positions, and they may, generally, perform a similar function. [4,27,107,108]. The first cluster is referred to as cluster A. This cluster accommodates eight gene loci and is situated on the long arm of chromosome 12 (12L) [13]. The second cluster is regarded as cluster B. This cluster harbours nine genes, and it is positioned on the short arm of chromosome 4 (4S) [13,27].

The third cluster is called cluster C. This cluster is sited on the short arm of chromosome 6 (6S) and possesses four genes [27]. The fourth cluster is considered as cluster D. This cluster contains five genes/QTLs, and it is confined to chromosome 4L (27,31]. The fifth cluster, which is cluster E, harbours four gene loci, and it is situated on chromosome 3L [27,30].

The *BPH1*-resistant locus was placed on chromosome 12 at the interval of two SSR markers, RM28366 and RM463 [32]. In the other two investigations using different RFLP/AFLP markers [73,76], the *BPH1* locus was positioned on chromosome 12L. Sun et al. [68] revealed that the *BPH2* gene locus was placed on chromosome 12L, harboured by two flanking SSR markers, RM7102 and RM463. In a similar finding employing RFLP markers [63], the *BPH2* locus was detected on chromosome 12. Jairin et al. [69] revealed that the *BPH3* locus was designated on chromosome 6S at the interval of two SSR markers (RM589 and RM588). The *BPH4* locus was found in an indica cultivar, Babawee, the locus was positioned on chromosome 6S and harboured by two flanking markers, RM589 and RM586 [64]. The *bph5* locus present in the ARC10550 rice cultivar (Table 3) is an aggregation of many QTLs (*qBphNp(48h)-1*, *qBphDs-6*, *qBphDw(30)-8*, *qBphDw(30)-3* and *qBphNp(72h)-12*) detected on distinct rice chromosomes (1, 3, 6, 8 and 12). These QTLs collectively accounted for 55.6% of the phenotypic difference for resistance in TN1 x ARC10550 progenies [105]. In Swarnalata [38], *BPH6* gene was designated on chromosome 4L between two SSR markers, RM6997 and RM5742. The *BPH7* locus (formerly named *bph7*) was sourced from an indica cultivar, T12; the locus was found on chromosome 12L at the interval of two SSR markers (RM28295 and RM313). Further genetic analysis, with more precision, revealed that *BPH7* was located between two SSR markers (RM3448 and RM313) region. This region is 150 and 300 kb in *Nipponbare* and 93-11 genomes, respectively [109]. A major resistant gene, *Bph9*, found in rice cultivar Karahamana was placed on chromosome 12 at the interspace of two SSR markers, RM463 and RM5341 [118]. In a similar experiment adopting RAPD and RFLP markers in cultivar Pokkali, the *Bph9* gene was mapped between two markers (OPRO4 and S2545) on chromosome 12L [75]. *Bph10* locus in *O. australiensis* was discovered on chromosome 12L [68]. In *O*. *officinalis*, the *Bph-10* locus was identified on chromosome 12 between SSR and RFLP markers, RM260 and RG157L-B, respectively [119]. The recessive locus, *bph11(t)*, found in *O. officinalis*, was situated on chromosome 3L [117]. The *BPH12* (previously *Bph12(t)*) locus derived from *O. latifolia*, flanked by two SSR markers (RM16459 and RM1305), was found on chromosome 4S [90]. In *O*. *officinalis*, the *Bph13(t)* locus was mapped on chromosome 3S using RAPD and STS markers [62]. On the contrary, Liu et al. [120] disclosed that the *Bph13*(t) locus was placed on chromosome 2L, flanked by two SSR markers, RM240 and RM250.

Huang et al. [113] revealed that *Bph14* gene was mapped between two RFLP markers (R1925 and R2443) on chromosome 3L. On the other hand, *Bph15* locus was positioned on chromosome 4S at different RFLP marker intervals [113,121]. A minor locus, *bph16* (previously referred to as *bph12(t)*), found between two markers (RFLP), G271 and R93, was positioned at chromosome 4L [117]. The *Bph17* gene was found in Rathu Heenati on chromosome 4S at physical interspace of two markers (SSR), RM8213 and RM5953. This resistance gene alone contributed 83.9% of the phenotypic differences in the advanced resistance lines [70]. The *Bph18(t)*, found in *O. australiensis* [7], was mapped within the 843-kb region between two markers (R10289S and RM6869) and absolutely cosegregated with the 7312T4A marker on chromosome 12L. However, in a fine map, using a backcross population of Junam x IR65482, the *Bph18* gene was delineated to the 27-kb region through the *Nipponbare* genome sequence between two markers, BIM3 and BN162 [122]. A minor recessive genetic factor, *bph19(t),* was fine-mapped on chromosome 3S at the interval of two SSR markers (RM6308 and RM3134) [123]. The *Bph20(t)* locus was mapped on chromosome 4S at a physical distance of two SSR markers, MS10 and RM5953. This resistant locus was obtained from IR71033-121-15, an *O. minuta* introgression line. Similarly, *Bph21(t)* emerged from the IR71033-121-15 line; this locus was situated on chromosome 12L [110]. Hou et al. [124] revealed that *Bph22(t)* locus was positioned on chromosome 4, while *Bph23(t)* was mapped on chromosome 8. A minor resistance locus, *bph24*(t), was sourced from *O*. *rufipogon* with accession number 106,412 [125]. Two major genes, *Bph25* and *Bph26*, were derived from ADR52, an indica cultivar. The two genes were detected on chromosomes 6S and 12L, respectively [5]. On the other hand, the *Bph27*, a dominant genetic factor, was obtained from *O*. *rufipogon* Griff.; this locus was situated on chromosome 4L flanked by two SSR markers, RM16846 and RM16888. An additional linkage investigation based on the *Nipponbare* genome sequence revealed that the 86.3-kb region is the chromosomal position occupied by *BPH27* [116]. In addition, *Bph27* was previously found on chromosome 4L at the interval of two SSR markers, RM471 and RM273 [81]. Presently, *bph18*(t) has been renamed as *Bph27* [126], and the new nomenclature has been documented in the rice database (*Oryza* database; http://www.shigen.nig.ac.jp/rice/oryzabase/). The *Bph28*(t) locus was found at a 64.8-kb position on chromosome 11 between two InDel markers, InDel55 and InDel66 [78]. The *Bph29* gene was derived from *O*. *rufipogon* flanked by two InDel markers (BYL7 and BYL8) and was placed on chromosome 6S [79]. The *Bph30* locus was sourced from an indica cultivar, AC-1613. Initially, the locus was detected by QTL scan at the interval of two SSR markers (RM16278 and RM16425) on chromosome 4S. However, subsequent mapping analysis showed that *Bph30* is placed at a 37.5-kb region based on the *Nipponbare* genome, within the distance of two SSR markers, SSR-28 and SSR-69 [41].

The *BPH31* locus, derived from CR2711-76, was situated on chromosome 4L within a 475-kb space at the interval of two InDel markers (PA26 and RM2334) [30]. The *BPH32* gene, a major gene found in cultivar Pbt33, has been positioned on chromosome 4S between two markers (SSR), RM19291 and RM8072 [29]. Naik et al. [130] revealed that the *BPH33*(t) locus was mapped to chromosome 1 within the distance of two SSR markers, RM488 and RM11522. On other hand, the *Bph33* is a major QTL derived from two indica cultivars, KOLAYAL and POLAYAL; this locus was finely positioned on chromosome 4S between two InDel markers, H99 and H101 [28]. The *Bph34* is a dominant genetic factor obtained from *O. nivara*, an undomesticated rice species with accession number, IGRC104646; this locus was detected on chromosome 4L between two markers (SNP), AX-95952039 and AX-95921548. Apart from the SNP markers, RM16994 and RM17007 (SSR) are also closely associated with the locus [31]. The *Bph35* is a tolerant QTL found using QTL lciMapping at two InDel markers (PSM16 and R4M13) interspaced on chromosome 4. Additional fine-mapping analysis revealed that *Bph35* is finally delineated within the interspace of 6.28 to 6.93 Mb [34]. The *Bph36* is a major locus obtained from advanced lines RBPH16 and RBPH17 of GX2183, a wild rice. The locus was situated at a 38-kb region on chromosome 4S between two InDel markers, S13 and X48 [33]. The *BPH37*, a dominant QTL acquired from the KWQZ/IR64 population, was located on chromosome 1 at the interval of two markers, RM302 and YM35 [32]. Another major QTL, *BPH38*(t), originated from the HHZ/Khazar population, was positioned at chromosome 1L between two markers (SNP), 693,369 and id 10,112,165 [13].

## 6. Gene Manifestation of BPH Resistance Transgenic Plants

New genes that express resistance to BPH have been determined from other sources. This resulted in the evolvement of transgenic plants that administer BPH resistance. For instance, *Galanthus nivalis agglutinin* (GNA), which is a snow-drop lectin gene, manifests virulence or antibiosis to BPH when fed or administered with synthetic feed [131]. Rice lines harbouring the transgene (GNA) showed BPH resistance triggered by two promoter genes: rice sucrose synthase (*RSs1*gene), which is a phloem-specific promoter, and the maize ubiquitin *ubil* gene, a constitutive promoter. These transgenes were incorporated into the rice plant progenies, as testified by Southern blot and PCR analyses. The result of Western blot analysis indicated that 2% GNA was recorded out of the whole protein from some of the tested transgenic plants. Feeding and insect bioassay analyses revealed that the GNA gene present in transgenic plants reduces the progeny production (fecundity) and survival rate of insects, restricts insect growth and development, and inhibits BPH’s feeding process. GNA is the first transgene that was described to have detrimental effects on BPH in rice plants [4]. In another investigation, Ren et al. [29] reported that transgenic incorporation of *Bph32* into a BPH-sensitive rice variety considerably enhances resistance to BPH. Additional phenotyping showed that *Bph32* is substantially manifested in the leaf sheath, which BPH normally occupies and uses as nourishment. This result further indicates that *Bph32* may confer feeding inhibition to BPH. The Western blot test detected the existence of Pph (Ptb33) and Tph (TN1) proteins, employing a Penta-His antibody, and the two proteins were indissoluble. The transgenic plants of the *BPH18* promoter gene, *beta-glucuronidase* (GUS), showed great GUS appearance in the conducting tissues, particularly the phloem tissue of the leaf sheath, which BPH colonises for food from the host [122].

## 7. Cloning of BPH Resistance Genes

Successes have been accomplished in the cloning of some genes that govern BPH resistance [29,37,74,79,122,132,133,134]. *Bph6* yields an unspecified protein that is peculiar to exocysts and is related to the exocyst’s smaller units (OsEXO70E1; Table 4). The *Bph6* appearance enhances exocytosis and confers cell wall rigidity and maintenance. In plants possessing the *Bph6* gene, a well-coordinated pathway is triggered for cytokinin, jasmonic and salicylic acids, which manifests a wide resistance spectrum to entire BPH races as well as the white-backed planthopper [134]. The *BPH9* gene produces a seldom-occurring nucleotide-binding and leucine-rich (NLR)-containing protein found on the inner membrane system, which results in the death of cell phenotypes. It triggers jasmonic and salicylic acid signaling routes in rice plants and thus administers resistance to BPH (i.e., antixenosis and antibiosis) [133]. The *Bph14* gene provides a coiled-coil, nucleotide-binding and leucine-rich repeat (CC-NB-LRR) protein, which resists BPH attack at juvenile and maturity growth phases. It was first isolated through positional cloning, and, according to two-parent sequence comparison analysis, it contains a special LRR protein that might play a role in the sensing of BPH attacks. This process activates the host–defence response mechanism, perhaps by means of stimulation of salicylic acid-dependent resistance pathways. This further triggers the deposition of callose within the phloem tissue and that overcomes the BPH attack on its host [37]. The *Bph17* gene is a cluster of genes that compose of several sequential repeated genes (*OS04g0201900*, *Os04g0202350*, *Os04g0202500* and *Os040g020800*). The four genes yield putative lectin receptor kinases, referred to as *Oryza sativa* Lectin Receptor Kinase (OsLecRK), and the cluster includes OsLecRK1, OsLecRK2, OsLecRK3 and OsLecRK4 [132].

Further genetic analysis of *Bph17* gene confirmed that the OsLecRK1–OsLecRK3 is the major gene cluster that administers BPH resistance to a high degree. In other words, these genes accord a wide resistance spectrum to BPH [132].

The *Bph18* gene, yields the CC-NBS-NBS-LRR protein, with double NBS domains, in contrast with most resistance genes of rice, which carry a single NBS domain [122]. The proteins of *Bph18* are mainly restricted to the membrane-bound organelles in cells such as the Golgi apparatus, endoplasmic reticulum, trans-Golgi network and prevacuolar chambers, therefore indicating that the gene may detect BPH invasion at endomembrane levels in phloem tissue. In a genome comparison analysis of near-isogenic lines (NILs) of *Bph18* and *Bph26*, the result showed that *Bph18* is positioned at an identical locus with *Bph26*. In spite of that, these two genes possess a distinct DNA sequence. NILs of the two genes exhibited contrasting BPH reaction with a varied display pattern of host plant defence-regulated genes, hence, revealing that the two genes perform different functions [122]. The *Bph26* gene, just like most resistant genes of rice, supplies the CC-NBS-LRR protein, which confers sucking resistance in the phloem sieve element (antibiosis) of the host against BPH invasion. In a DNA sequence comparison analysis of the eating behaviour of BPH virulent biotype 2, the result revealed that *Bph26* is similar to the *BPH2* resistant locus [74]. *Bph29* is a recessive gene that manufactures a B3 DNA-binding domain-containing protein associated with conducting tissues, where BPHs attack. The gene regulates the process of the salicylic acid signalling route and represses the jasmonic acid/ethylene-dependent route in response to BPH attack, thereby inducing the deposition of callose in phloem tissue, leading to BPH resistant of the host plant [79]. The *BPH32* gene synthesises an unknown short consensus repeat (SCR) protein domain that administers BPH resistance through antibiosis. It is found on the outer membrane of the rice plant cell and is largely predominant in the leaf sheath, a place where BPHs menace and feeding initiates [29].

## 8. Conclusions

The enhancement of rice production faces serious threats due to frequent population increase, detrimental consequences of climate change, as well as the evolution of new BPH biotypes. The use of resistant varieties remains the most economically viable and environmentally friendly strategy that reduces attacks by BPHs and, ultimately, improves rice productivity. The majority of the BPH-resistant genes detected do not accord broad resistance spectrum to various BPH populations/biotypes. Therefore, the evolution of new rice varieties with a wide resistance spectrum to BPH populations from diverse genetic sources is essential to overcome the resistance of new BPH biotypes. This can be accomplished through the adoption of marker-assisted gene pyramiding. This strategy expedites the processes of varietal improvement and, finally, ensures resistance that is more durable. So far, above 37 genes/QTLs that control BPH resistance have been established through indica and wild species, and eight of them have been successfully cloned. Additionally, five gene clusters have been identified from the previous investigations. It is our hope that many more genes and sources of resistance will be explored in due course.

## Figures and Tables

**Table 1:** Some brown planthopper (BPH)-resistant donor varieties used in many rice breeding programme and their mechanisms of resistance.

Gene/QTL Designation	Variety	Gene Function	BPH Reaction/Phenotype	Reference
*Bph9*	Balamawee	-	R	[4]
*Bph6*	Swanalata	Antibiosis	R	[42]
*Bph7*	T12	Antibiosis, tolerant	HR	[42]
*BPH2*, *BPH3*	Ptb33	Antibiosis	R	[42]
*Bph17, Bph3*	Rathu Heenati	Antibiosis, antixenosis	R	[14,20]
*Bph8*	Chin Saba	-	HS	[42]
*Bph9*	Pokkali	Antibiosis, tolerant	MR	[42]
*Bph18*	*O. australiensis* (Acc, No. 100882)	-	R	[4]
*BPH2*	ASD 7	Antibiosis, tolerant	R	[42]
*Bph6, Bph3*	*O. officinalis* (Acc. No. 100896)	-	R	[4]
*BPH2*	IR36	Antibiosis, tolerant	MR	[20,42]
*BPH4*	Babawee	Antibiosis, antixenosis	R	[4,20,42]
*BPH1*	Mudgo	Antixenosis, antibiosis	R	[20,43]
*BPH1, BPH37*	IR64	Antibiosis, tolerant	MR	[32,42]
*BPH30*	AC-1613	Antibiosis	R	[41]
*BPH31*	CR2711	Antibiosis, antixenosis, tolerance	R	[30]
*BPH32*	Ptb33	Antibiosis	R	[29]
*BPH33*	KOLAYAL, PPLIYAL	Antibiosis, antixenosis	R	[28]
*BPH36*	GX2183	Antibiosis, antixenosis	R	[33]

HR = highly resistant; R = resistant; MR = moderately resistant; HS = highly susceptible.

**Table 2:** List of some BPH resistance genes/quantitative trait loci (QTLs) and their clusters.

Gene/QTL Cluster	Gene/QTL Designation	Chromosomal Position	Reference
A	*Bph1*	12L	[76]
	*Bph2*	12L	[68]
	*Bph7*	12L	[109]
	*Bph9*	12L	[75]
	*Bph10*	12L	[77]
	*Bph18*	12L	[7]
	*Bph21*	12L	[110]
	*Bph26*	12L	[5]
B	*QBph4.1*	4S	[111]
	*qBph4.2*	4S	[112]
	*Bph12*	4S	[90]
	*Bph15*	4S	[113]
	*Bph17*	4S	[70]
	*Bph20*(t)	4S	[110]
	*Bph30*	4S	[41]
	*Bph33*	4S	[28]
	*Bph36*	4S	[33]
C	*BPH3*	6S	[84]
	*BPH4*	6S	[64]
	*Bph25*	6S	[5]
	*Bph29*	6S	[79]
D	*Bph6*	4L	[38]
	*Bph12(t)*	4L	[114]
	*Bph27(t)*	4L	[115]
	*Bph27*	4L	[116]
	*Bph34*	4L	[31]
E	*QBph3*	3L	[111]
	*bph11(t)*	3L	[117]
	*Bph14*	3L	[37]
	*Bph31*	3L	[30]

**Table 3:** List of some successfully mapped BPH resistance genes/QTLs.

Gene/QTL	Chr. no.	Marker Type	Functional/Flanking Marker Name	Donor Parent	Rice Type	Reference
*BPH1*	12	SSR	RM28366-RM463	IR64	Indica	[32]
*BPH1*	12L	RFLP, AFLP	cm5814N-cm2802N, pBPH4-pBPH14	Mudgo	Indica	[24,43,76]
*BPH1*	12	RFLP	XNpb248-XNpb336	TKM 6	Indica	[73]
*BPH2*	12	RFLP	G2140	IR1154-243	Indica	[63]
*BPH2*	12L	SSR	RM7102-RM463	ASD 7	Indica	[68]
*BPH3*	6S	SSR	RM589-RM588, RM190, RM19291-RM7082	Rathu Heenati, Ptb33	Indica	[69,84]
*Qbph3*	3	SSR	RM313-RM7	Rathu Heenati	Indica	[70]
*QBph3*	3L	-	C3-14	*O. officinalis*	Wild	[111]
*BPH4*	6S	RFLP, SSR	C76A, RZ516, R1954, RZ588, R2147, R2171, C76B, RM225-RM217, RM589-RM586	Babawee	Indica	[64,127]
*QBph4.1*	4S	-	C3-14	*O. officinalis*	Wild	[111]
*qBph4.2*	4	-	RM261-S1, XC4-27	*O. australiensis*	Wild	[112]
*bph5*	-	-	-	ARC10550	Indica	[105]
*qBphNp(48h)-1*	1L	SSR	RM11704-RM1068	ARC10550	Indica	[105]
*qBphDw(30)-3*	3L	SSR	RM7179-RM6987	ARC10550	Indica	[105]
*qBphDs-6*	6S	SSR	RM547-RM5855	ARC10550	Indica	[105]
*qBphDw(30)-8*	8S	SSR	RM547-RM22741	ARC10550	Indica	[105]
*qBphNp(72h)-12*	12L	SSR	RM27971-RM28024	ARC10550	Indica	[105]
*BPH6*	4L	SSR, STS	RM6997-RM5742, Y19-Y9	Swarnalata	Indica	[38]
*BPH7*	12L	SSR	RM3448-RM313	T12	Indica	[109]
*bph8(t)*	-	-	-	Col. 5, Col. 11	Thailand	[60]
*bph8(t)*	-	-	-	Chin Saba	Myanmar	[60]
*Bph9*	12L	RAPD, RFLP	OPRO4-S2545	Pokkali	Indica	[75]
*Bph9*	12	SSR	RM463-RM5341	Karahamana	Indica	[118]
*Bph-10*	12	SSR, RFLP	RM260-RG157L-B	*O*. *officinalis*	Wild	[119]
*Bph10*	12L	RFLP	RG457	*O. australiensis*	Wild	[68]
*Qbph10*	10	SSR	RM484-RM496	Rathu Heenati	Indica	[70]
*bph11(t)*	3L	-	-	*O*. *officinalis*	Wild	[117]
*Qbph11*	11	RFLP	XNpb202-C1172	DV85	Indica	[128]
*Bph12*	4S	SSR, RFLP	RM16459-RM1305, RM335, RM261, RM185, C820, R288, C946	*O. latifolia*	Wild	[90,129]
*Bph13(t*)	3S	RAPD, STS	AJ09b_230_, AJ09c	*O. officinalis*	Wild	[62]
*Bph13(t)*	2L	SSR	RM240-RM250	*O*. *eichingeri*	Wild	[120]
*Bph14*	3L	RFLP	R1925-R2443, R1925-G1318	*O*. *officinalis*	Wild	[37,113]
*Bph15*	4S	RFLP	C820-R288, C820-S11182, RG1-RG2, M1	*O*. *officinalis*	Wild	[113,121]
*bph16*	4L	RFLP	G271-R93	-	-	[117]
*Bph17*	4S	SSR	RM8213-5953	Rathu Heenati	Indica	[70]
*Bph18*	12L	STS, SSR	*R10289S*-*RM6869*, *BIM3*-*BN162*	*O. australiensis*	Wild	[7,122]
*bph19(t)*	3S	SSR	RM6308-RM3134	AS20-1	Indica	[123]
*Bph20(t)*	4S	SSR	MS10-RM5953	*O. minuta*	Wild	[110]
*Bph21(t)*	12L	SSR	RM3726-RM5479	*O. minuta*	Wild	[110]
*Bph22(t)*	4	SSR	RM8212-RM261	*O. rufipogon*	Wild	[124]
*Bph23(t)*	8	SSR	RM2655-RM3572	*O. rufipogon*	Wild	[124]
*bph24(t)*	-	-	-	*O. rufipogon*	Wild	[125]
*Bph25*	6S	SSR	S00310-RM8101	ADR52	Indica	[5]
*Bph26*	12L	SSR, InDel, SNP	RM3331-S20103, RM5479, RM28449-RM3813, DS-72B-DS-173B, ID-23-4-ID-174	ADR52	Indica	[5,74]
*BPH27*	4L	SSR	RM16846-RM16888	*O. rufipogon* Griff	Wild	[116]
*Bph27(t)*	4L	InDel	Q52-Q20	Balamawee	Indica	[115]
*Bph28(t)*	11L	InDel, SSR	Indel55-Indel66, RM202-RM5961	DV85	Indica	[78]
*BPH29*	6S	InDel	BYL7-BYL8	*O. rufipogon* Griff	Wild	[79]
*BPH30*	4S	SSR	SSR-28-SSR-69, RM16278-RM16425, RM16294, RM16299	AC-1613	Indica	[41]
*BPH31*	3L	InDel	PA26-RM2334	CR2711-76	Indica	[30]
*BPH32*	6S	SSR	RM19291-RM8072	Ptb33	Indica	[29]
*BPH33*	4S	InDel	H25-D17, H14-H84, H99-H101	KOLAYAL, POLIYAL	Indica	[28]
*BPH33(t)*	1	SSR	RM488-RM11522	Velluthacheera	Indica	[130]
*BPH34*	4L	SNP, SSR	AX-95952039, AX-95921548, RM16994, RM17007	*O*. *nivara*	Wild	[31]
*BPH35*	4	InDel	PSM16-R4M13, PSM19, PSM20	*O. rufipogon*	Wild	[34]
*BPH36*	4S	InDel	S13-X48	*O*. *rufipogon* Griff	Wild	[33]
*BPH37*	1	SSR, InDel	RM302-YM35	IR64	Indica	[32]
*BPH38(t)*	1L	SNP	693,369, id 10,112,165	Khazar	Indica	[13]

**Table 4:** Successfully cloned BPH resistance genes.

Name of Gene	Position on Chromosome	Characteristics	Year of Cloning	Donor Parent	Rice Type	Mapping Region	Reference
*BPH6*	4L	Exocyst-localised protein	2018	Swarnalata	Indica	18.1-kb	[134]
*BPH9*	12L	CC-NBS-NBS-LRR	2016	Pokkali	Indica	47-kb	[133]
*Bph14*	3L	CC-NB-LRR	2009	*O. officinalis*	Wild	34-kb	[37]
*Bph17*	4S	OsLecRK1- OsLecRK4	2014	Rathu Heenati	Indica	79-kb	[132]
*Bph18*	12L	CC-NBS-NBS-LRR	2016	*O. australiensis*	Wild	27-kb	[122]
*Bph26*	6S	CC-NB-LRR	2014	ADR52	Indica	135-kb	[74]
*bph29*	6S	B3 DNA binding protein	2015	*O. rufipogon*	Wild	24-kb	[79]
*BPH32*	6S	SCR domain containing protein	2016	Ptb33	Indica	190-kb	[29]
